# Lung carcinogenicity of inhaled multi-walled carbon nanotube in rats

**DOI:** 10.1186/s12989-016-0164-2

**Published:** 2016-10-13

**Authors:** Tatsuya Kasai, Yumi Umeda, Makoto Ohnishi, Takashi Mine, Hitomi Kondo, Tetsuya Takeuchi, Michiharu Matsumoto, Shoji Fukushima

**Affiliations:** Japan Bioassay Research Center, Japan Organization of Occupational Health and Safety, 2445 Hirasawa, Hadano, Kanagawa 257-0015 Japan

**Keywords:** Lung carcinoma, Lung carcinogenicity, Multi-walled carbon nanotube, MWCNT, MWNT-7, Inhalation carcinogenicity, Whole body inhalation, Rat

## Abstract

**Background:**

Multi-walled carbon nanotubes (MWCNTs) constitute one of the most promising types of nanomaterials in industry today. With their increasing use, the potential toxicity and carcinogenicity of MWCNT needs to be evaluated in bioassay studies using rodents. Since humans are mainly exposed to MWCNT by inhalation, we performed a 104-week carcinogenicity study using whole-body inhalation exposure chambers with a fibrous straight type of MWCNT at concentrations of 0, 0.02, 0.2, and 2 mg/m^3^ using male and female F344 rats.

**Results:**

Lung carcinomas, mainly bronchiolo-alveolar carcinoma, and combined carcinomas and adenomas were significantly increased in males exposed to 0.2 and 2 mg/m^3^ MWNT-7 and in females exposed to 2 mg/m^3^ MWNT-7 compared to the clean air control group. However, no development of pleural mesothelioma was observed. Concentration-dependent toxic effects in the lung such as epithelial hyperplasia, granulomatous change, localized fibrosis, and alteration in BALF parameters were found in MWNT-7 treatment groups of both sexes. There were no MWNT-7-specific macroscopic findings in the other organs, including the pleura and peritoneum. Absolute and relative lung weights were significantly elevated in male rats exposed to 0.2 and 2 mg/m^3^ MWNT-7 and in all exposed female groups. The lung burdens of MWNT-7 were clearly increased in a concentration-dependent as well as a duration-dependent manner.

**Conclusion:**

There is clear evidence that MWNT-7 is carcinogenic to the lungs of male and female F344 rats, however no plural mesothelioma was observed.

**Electronic supplementary material:**

The online version of this article (doi:10.1186/s12989-016-0164-2) contains supplementary material, which is available to authorized users.

## Background

One of the most important developments in industrial technology is nanotechnology. Various nanomaterials, especially carbon nanotubes (CNTs), have exceptional electrical, mechanical, and thermal properties, enabling the commercialization of various types of CNTs for use with numerous applications in industry, and in recent years the production of CNTs has significantly increased [1]. On the other hand, with the rapid growth of CNT use, serious concerns have been expressed about their adverse effects on the health of workers during CNT manufacturing and handling processes and on the health of consumers exposed to commercial end products containing CNTs. At present, however, neither epidemiological nor medical case studies have been reported on health consequences in CNT-exposed workers or consumers.

Long, straight types of multi-walled carbon nanotube (MWCNT) fibers, like asbestos, have a high aspect ratio and are able to persist in biological tissues [2, 3]. The “Stanton hypothesis” [4, 5], also known as the fiber paradigm [6], asserts that fibers longer than 8 μm and less than 0.25 μm in diameter have high carcinogenic potential. High aspect ratio fibers (especially fibers having diameters less than 3 μm and lengths of greater than 5 μm, L/D ratio > 3) are thought to play an essential role in the induction of intrathoracic tumors by fibrous materials [2, 6–9]. Poland et al. reported that intraperitoneal administration in rodents of long, straight types of asbestos or MWCNT fibers, but not nanoparticulate carbon black or short asbestos fibers or short or tangled MWCNT, resulted in asbestos-like pathogenic responses [10]. Thus, there is obvious reason for concern for possible asbestos-like health effects, such as induction of pulmonary carcinoma and mesothelioma, by exposure to fibrous MWCNTs. Importantly, Takagi et al. [11] demonstrated that peritoneal mesotheliomas were induced by intraperitoneal administration of one type of fibrous MWCNT (MITSUI MWCNT-7, MWNT-7 in the present study) to p53 knockout mice. Subsequently, several studies reported that malignant mesotheliomas developed in mice or rats after intraperitoneal or intrascrotal administration of MWNT-7 [2, 12–14]. Thus, MWNT-7 is carcinogenic to the mesothelium upon direct exposure.

MWCNT fibers are very light and easily become airborne, making inhalation the primary route of human exposure [15, 16]. To assess the risk of MWCNT to exposed workers, *in vivo* toxicity studies in rodents exposed to MWCNT via inhalation are crucial. Ryman-Rasmussen et al. [17] and Porter et al. [18] reported that MWCNT inhaled by mice is toxic to lung and plural tissues, and Sargent et al. [19] reported that inhalation of MWCNT at 5 mg/m^3^ for 15 days followed by a 17 month postexposure period promoted methylcholanthrene initiated lung-carcinogenesis in mice. Taken together, these results show that airborne long straight MWCNT fibers are potentially carcinogenic to mouse lung and pleural tissues.

We have developed a dry aerosol generation and exposure system (cyclone sieve method) for whole-body inhalation exposure to MWCNTs. Using this method, we conducted 6-h, 2-week, and 13-week inhalation studies of MWNT-7 using rats [20–22]. The results revealed persistent inflammation and granulomatous changes with multinucleated giant cells in the lung, a concentration-dependent increase in the deposition of MWNT-7 in the lung, and the presence of MWCNT-containing alveolar macrophages. The concentration-dependent increase in the retention of MWNT-7 in the lung was associated with the severity of toxicity and was particularly apparent in the 13-week study [22]. Importantly, MWNT-7 fibers were also observed in the subpleural area and diaphragm. These results indicate that inhaled MWNT-7 is deposited deep into the respiratory tract and can be translocated into the pleural cavity, and that exposure to MWNT-7 induces pathological changes in the lung and chest cavity.

Taken together, the results of all of the studies cited above led to the hypothesis that inhalation of MWNT-7 aerosol may result in tumor development in the lung and possibly the pleura. Therefore, in order to clarify the carcinogenicity of MWNT-7, we conducted a 2-year carcinogenicity study of MWNT-7 by whole body inhalation based on a widely accepted protocol [23]. In this study, F344 male and female rats, 6-weeks-old at the commencement of the study, were exposed to MWNT-7 aerosol for 6 h/day, 5 days/week for 104 weeks at concentrations of 0, 0.02, 0.2, and 2 mg/m^3^ using our dry aerosol generation and exposure system.

## Results

### Environmental conditions of the inhalation chambers

All inhalation chambers were maintained at an air exchange rate of 10 times/h (1668–1671 l/min), a temperature of 22.9–23.0 °C, and a humidity of 52.7–53.8 % during each exposure period (Table 1).

**Table 1:** Characterization of chamber environment

Target concentration (mg/m^3^)	0	0.02	0.2	2
Temperature (°C)	23.0 ± 0.2	22.9 ± 0.1	23.0 ± 0.2	23.0 ± 0.2
Humidity (%)	53.8 ± 1.8	52.9 ± 2.4	53.3 ± 2.1	52.7 ± 2.0
Chamber air flow (L/min)	1670 ± 37	1671 ± 36	1671 ± 37	1668 ± 38
Mass concentration (mg/m^3^) ^a^	0.000 ± 0.000	0.020 ± 0.001	0.204 ± 0.014	2.018 ± 0.069
MMAD (GSD): 1 week ^b^	-	1.3 μm (3.0)	1.4 μm (2.9)	1.3 μm (2.6)
MMAD (GSD): 14 week	-	1.3 μm (2.8)	1.3 μm (2.9)	1.3 μm (2.9)
MMAD (GSD): 27 week	-	1.3 μm (2.9)	1.3 μm (2.9)	1.3 μm (2.8)
MMAD (GSD): 40 week	-	1.3 μm (2.9)	1.3 μm (2.9)	1.3 μm (2.9)
MMAD (GSD): 53 week	-	1.4 μm (3.0)	1.4 μm (2.9)	1.4 μm (2.9)
MMAD (GSD): 66 week	-	1.2 μm (2.9)	1.3 μm (2.9)	1.4 μm (2.8)
MMAD (GSD): 79 week	-	1.3 μm (2.9)	1.3 μm (2.9)	1.4 μm (2.8)
MMAD (GSD): 92 week	-	1.3 μm (3.0)	1.3 μm (3.0)	1.4 μm (2.8)
MMAD (GSD): 102 week	-	1.4 μm (2.9)	1.3 μm (2.7)	1.3 μm (2.9)

Values indicates means ± SD

^a^ The mass concentrations were calibrated from the particle concentration of MWNT-7 in the inhalation chamber measured by the OPC

^b^ MMAD: Mass median aerodynamic diameter, GSD: geometric standard deviation

### MWNT-7 concentrations and particle size distributions

**Fig. 1:**
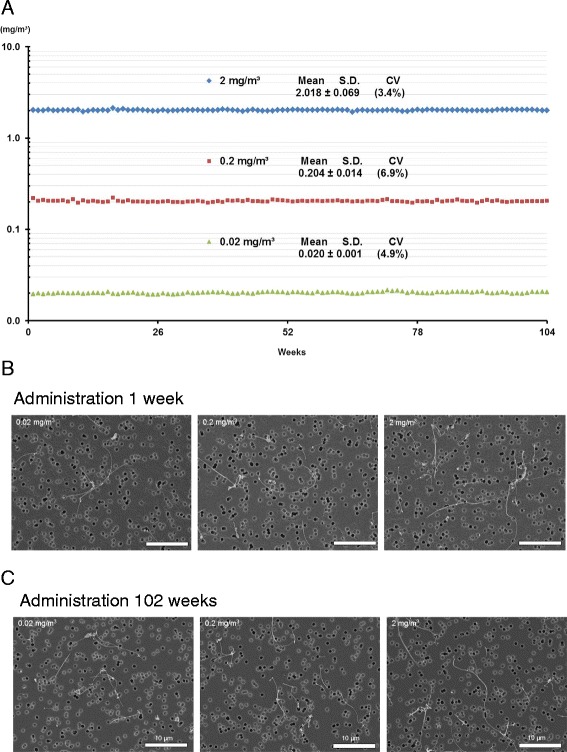
**a** MWNT-7 concentrations in the inhalation chambers during the 104-week experimental period. Concentrations of MWNT-7 particles in the inhalation chamber were monitored by the optical particle controller throughout each 6 h exposure period during the 104 weeks of the study. Mass concentrations (mg/m^3^) of MWNT-7 in the chambers was calibrated by multiplying the CPM by the mass per particle (see Methods for details). The target concentrations of MWNT-7 were 0.02, 0.2, and 2 mg/m^3^; panel A shows the measured concentrations of MWNT-7 aerosols in the chambers. **b** and **c** SEM images of MWNT-7 in the inhalation chamber. SEM images of MWNT-7 in the chamber are shown in panels **b** and **c**. The fibers shown in the figure were collected on a polycarbonate filter at 1-week and 102 weeks. The MWNT-7 aerosol was in the form of individual and fibers in all groups. Bar, 5 μm

MWNT-7 aerosols were freshly generated during each exposure period, and the MWNT-7 concentrations in the inhalation chambers were at the target doses throughout the 104-week experimental period: 0.020 ± 0.001 (mean ± SD) mg/m^3^ for the 0.02 mg/m^3^ group, 0.204 ± 0.014 mg/m^3^ for the 0.2 mg/m^3^ group, and 2.018 ± 0.069 mg/m^3^ for the 2 mg/m^3^ group. Figure 1a shows the concentrations of the MWNT-7 aerosols in the inhalation chambers throughout the experimental period. Mass median aerodynamic diameters (MMAD) and geometric standard deviations (GSD) of the MWNT-7 aerosol were in the range of 1.2–1.4 μm and 2.6–3.0 in all MWNT-7-exposed groups (Table 1). Scanning electron microscope (SEM) of MWNT-7 collected on polycarbonate filters demonstrated that most MWNT-7 s were single straight fibers and were not aggregated, and no differences in MWNT-7 shape were found between the three target concentrations in any of the samples collected. Typical images of samples collected on weeks 1 and 102 are shown in Fig. 1b and c.

### Clinical observations, urinary, hematological and blood biochemical analyses

**Fig. 2:**
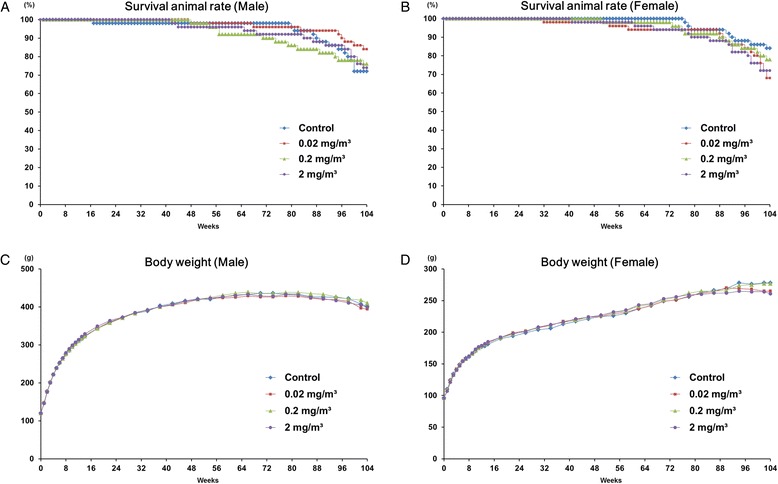
**a**, **b** Survival rates of rats exposed to MWNT-7 or clean air for 104 weeks. The survival rates of the rats are illustrated in panels A (*male*) and B (*female*). The survival rate exceeded 72 % in males and 68 % in females at the end of the 104 weeks experimental period. There were no differences in survival rates between the exposed and control groups. **c**, **d** Growth curves of rats exposed to MWNT-7 or clean air for 104 weeks. The growth curves of the rats are illustrated in panels **c** (*male*) and **d** (*female*). Body weights were measured once a week for the first 14 weeks and every 4 weeks thereafter. There was no growth retardation in any of the groups

Neither MWNT-7- related deaths nor clinical signs were observed in any MWNT-7-exposed male or female animals during the 104-week experimental period. Survival rates and body weight curves of male and female rats from the MWNT-7 and control groups are shown in Fig. 2. There were no differences between control and exposure group survival rates of either male or female rats; the survival rates exceeded 72 % and 68 % at the end of 104-week experimental period for males and females, respectively (Table 2). No growth retardation was found in any of the male or female groups during the experiment. The relative body weights of the 0.02, 0.2, and 2 mg/m^3^-exposed animals at the end of experiment were 98, 102, and 100 % for males and 95, 100, and 94 % for females, respectively, compared to their respective controls (Table 2).

**Table 2:** Absolute and relative, body weights and lung weights in rats exposed to MWNT-7 for 104 weeks

	Male				Female			
Group (mg/m^3^)	0	0.02	0.2	2	0	0.02	0.2	2
Numbers of animals examined	50	50	50	50	50	50	50	50
Survival animal number	36	42	38	37	42	34	39	36
(rate:%)	(72)	(84)	(76)	(74)	(84)	(68)	(78)	(72)
Body weight								
Terminal body weight (g) mean	401	394	410	399	278	265	277	261 *
SD	45	37	39	38	31	26	23	26
Relative body weight (%) mean	100	98	102	100	100	95	100	94
Lung weight								
Terminal lung weight (g) mean	1.31	1.34	1.51 **	2.37 **	0.90	0.94 **	1.14 **	1.87 **
SD	0.10	0.12	0.13	0.31	0.21	0.10	0.27	0.18
Relative lung weight (%) mean	0.36	0.37	0.40 **	0.65 **	0.35	0.38 **	0.45 **	0.79 **
SD	0.06	0.05	0.06	0.20	0.10	0.05	0.14	0.16

Terminal body weight (g): Absolute body weight at the end of 104 weeks

Relative body weight (%): Percentage of the body weight in the control group

Terminal lung weight (g): Absolute lung weight at the end of 104 weeks

Relative lung weight (%): Percentage of the terminal lung weight in each animal’s body weight

Significant difference; *: *p*≦0.05 **: *p*≦0.01 by Dunnett’s Test

Urinary, hematological, and blood biochemical analyses revealed no toxicological changes in either male or female rats exposed to 0.02, 0.2, or 2 mg/m^3^ compared with their respective controls (data not shown).

### Macroscopic findings and organ weights

**Fig. 3:**
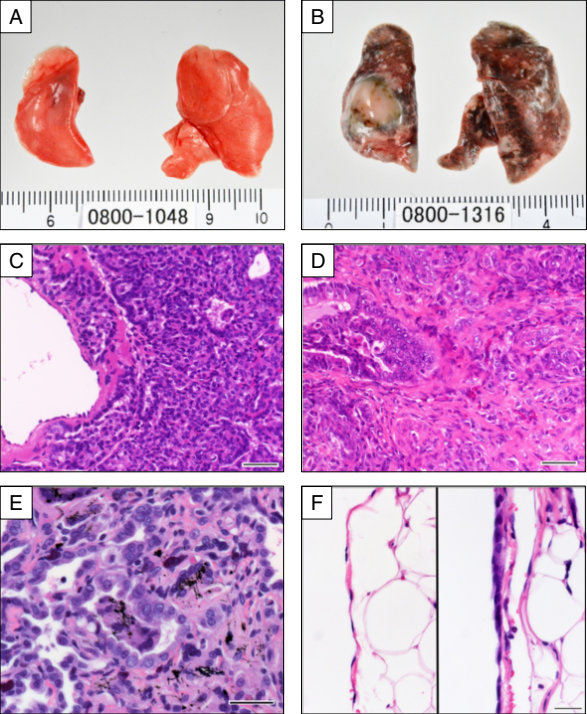
Macroscopic and microscopic findings of the lungs and peritoneal pleura. **a** Lung from a control male rat. **b** Multiple white areas and nodules in the lungs of a 2 mg/m^3^ MWNT-7-exposed male. **c** Bronchiolo-alveolar carcinoma in the lung of the male rat exposed to 2 mg/m^3^ MWNT-7. Carcinoma cells invaded the wall of the vein. Bar, 50 μm. **d** Bronchiolo-alveolar carcinoma in the lung of a female rat exposed to 2 mg/m^3^ MWNT-7. Proliferation of carcinoma cells accompanied by fibrous connective tissue is causing the disappearance of the existing alveolar construction. Bar, 50 μm. **e** Atypical hyperplasia accompanied by proliferative fibrous connective tissue and MWNT-7 deposition in the lung of a female rat exposed to 2 mg/m^3^ MWNT-7. Epithelial cell polarity is poorly defined and the nuclei of the cells vary in size. Bar, 30 μm. **f** The left image shows the normal parietal pleura of a control male rat. The right image shows a simple mesothelial hyperplasia of the parietal pleura in a male rat exposed to 2 mg/m^3^ MWNT-7. Bar, 25 μm

At the terminal necropsy, multiple grayish, white areas and nodules were found in the lungs of a large number of male and female rats exposed to 2 mg/m^3^ MWNT-7. The color tone of the lung surface was darkened in line with the exposure concentration (Fig. 3a and b). There were no MWNT-7-specific macroscopic findings in the other organs, including the pleura and peritoneum of the animals that survived to the end of the experiment or the animals that died or were sacrificed before the end of the experiment.

Absolute and relative lung weights were significantly elevated in male rats exposed to 0.2 and 2 mg/m^3^ MWNT-7, and they were significantly increased in all exposed female groups (Table 2). No MWNT-7 exposure-related increases were found in the other organs.

### Microscopic findings

The results of histopathological examination of the lungs, peritoneum and pleura are shown in Table 3. The incidences of bronchiolo-alveolar carcinomas, total carcinomas (bronchiolo-alveolar carcinomas, adenosquamous carcinoma, adenocarcinoma and squamous cell carcinoma), and total carcinomas and/or adenomas in males exposed to 0.2 and 2 mg/m^3^ MWNT-7 and females exposed to 2 mg/m^3^ MWNT-7 were significantly increased compared with their respective control groups. One adenosquamous carcinoma was found in each of the 2 mg/m^3^-exposed male and female groups, and one poorly differentiated adenocarcinoma and one squamous cell carcinoma were found in the 2 mg/m^3^-exposed female group; these malignant tumors very rarely arise spontaneously: no such tumors were observed in 599 control males or 600 control females in previous 104-week inhalation carcinogenicity studies in rats conducted by our institute during the last 10 years. Most bronchiolo-alveolar tumors were single tumors, while adenosquamous carcinoma, poorly differentiated adenocarcinoma, and squamous cell carcinoma tended to be multiple tumors. Tumor cells of the bronchiolo-alveolar tumors destroyed the alveolar structure and invaded the walls of bronchioles and blood vessels (Fig. 3c). It was noteworthy that the MWNT-7-induced bronchiolo-alveolar carcinomas were often accompanied by proliferative fibrous connective tissue, which was not present in the spontaneous lung carcinoma that developed in the control male rat (Fig. 3d).

**Table 3:** Histopathological findings of the lung, peritoneum and pleura in rats exposed to MWNT-7 for 104 weeks

	Male				Peto test	Female				Peto test
Group (mg/m^3^)	0	0.02	0.2	2		0	0.02	0.2	2	
Number of animals examined	50	50	50	50		50	50	50	50	
Neoplastic lesions										
Lung										
Bronchiolo-alveolar carcinoma	1	1	8 ^#^	10 ^##^	↑↑	0	1	0	5 ^##^	↑↑
Adenosquamous carcinoma	0	0	0	1		0	0	0	1	
Poorly differentiated adenocarcinoma	0	0	0	0		0	0	0	1	
Squamous carcinoma	0	0	0	0		0	0	0	1	
Total carcinoma	1	1	8 ^#^	11 ^##^	↑↑	0	1	0	8 ^##^	↑↑
Bronchiolo-alveolar adenoma	1	1	7 ^#^	5		3	1	4	3	
Total adenoma and/or carcinoma	2	2	13 ^##^	16 ^##^	↑↑	3	2	4	11 ^#^	↑↑
Peritoneum										
Malignant mesothelioma	0	3	1	1		0	0	0	0	
Non-neoplastic lesions										
Lung										
Bronchiolo-alveolar hyperplasia	2 (1.5)	6 (1.3)	13 * (1.2)	22 ** (1.0)		3 (1.3)	3 (1.0)	8 (1.1)	12 * (1.1)	
Atypical hyperplasia	0	0	1 (1.0)	10 ** (1.3)		0	0	0	14 ** (1.1)	
Alveolar hyperplasia	0	2 (1.0)	13 ** (1.0)	41 ** (1.0)		1 (1.0)	1 (1.0)	6 (1.0)	41 ** (1.1)	
Bronchiolar hyperplasia	0	0	4 (1.0)	8 ** (1.0)		0 (1.0)	0 (1.0)	4 (1.0)	26 ** (1.0)	
Accumulation: alveolar macrophage	2 (1.0)	7 (1.0)	5 (1.0)	48 ** (2.0)		2 (1.0)	6 (1.0)	9 (1.0)	48 ** (1.8)	
Focal fibrosis: alveolar wall	0	2 (1.0)	43 ** (1.0)	48 ** (1.8)		0	3 (1.0)	44 ** (1.0)	49 ** (1.2)	
Granulomatous change	0	5 (1.0)	42 ** (1.0)	50 ** (1.9)		0	3 (1.0)	45 ** (1.0)	50 ** (1.8)	
Pleura										
Simple mesothelial hyperplasia	3 (1.0)	3 (1.0)	7 (1.0)	12 * (1.0)		3 (1.0)	2 (1.0)	6 (1.0)	10 (1.0)	
Focal fibrosis: parietal (diaphragm)	0	0	2 (1.0)	6 * (1.0)		0	0	0	3 (1.0)	
Focal fibrosis: ventral (lung)	0 (1.0)	2 (1.0)	4 (1.0)	19 ** (1.1)		0	2 (1.0)	2 (1.0)	20 ** (1.0)	
Inflammation: mediastinum	15 (1.0)	18 (1.0)	21 (1.0)	26 * (1.0)		17 (1.0)	17 (1.0)	16 (1.0)	19 (1.0)	
Inflammation: diaphragm	0	0	1 (1.0)	1 (1.0)		0	1 (1.0)	1 (1.0)	1 (1.0)	

Values indicate number of animals bearing the lesion

Values in parentheses are the average of severity grade indexes of the lesions in affected animals. The average of severity grade indexes are calculated with a following equation. 〔Σ(grade × number of animals with grade)〕÷number of affected animals. Grade: 1 = slight, 2 = moderate, 3 = marked, 4 = severe

Significant difference; *: *p*≦0.05, **: *p*≦0.01 by Chi-square Test, #: *p*≦0.05, ##: *p*≦0.01 by Fisher Exact Test, ↑: *p*≦0.05, ↑↑: *p*≦0.01 by Peto’s Test

No MWNT-7-related tumors were induced in the other organs including the pleural and peritoneal mesothelium.

Incidences of bronchiolo-alveolar hyperplasia and alveolar hyperplasia were significantly increased in male rats exposed to 0.2 and 2 mg/m^3^ MWNT-7 and in female rats exposed to 2 mg/m^3^ MWNT-7. Incidences of bronchiolar hyperplasia and atypical hyperplasia were significantly increased in male and female rats exposed to 2 mg/m^3^ MWNT-7. Atypical hyperplasia consisted of epithelial cells with atypia and was accompanied by development of proliferative fibrous connective tissue and alveolar macrophages with phagocytosed MWNT-7 (Fig. 3e). Alveolar hyperplasia was composed of localized proliferation of type II alveolar epithelial cells, accompanied by fractured alveolar macrophages in the alveolar space. Bronchiolar hyperplasia was composed mainly of proliferating ciliated columnar epithelial cells in the terminal bronchiole area. Significantly elevated incidences of granulomatous change and focal fibrosis of the alveolar wall were noted in male and female rats exposed to 0.2 and 2 mg/m^3^ MWNT-7. Focal fibrosis, characterized by thickening and increased collagen fibers, was often associated with the granulomatous changes. Alveolar macrophages, with phagocytosed MWNT-7 s accumulated in the alveolar spaces in male and female rats exposed to 2 mg/m^3^ MWNT-7.

In the pleura, incidences of simple mesothelial hyperplasia of the parietal pleura (Fig. 3f) and focal fibrosis of the parietal pleura side of the diaphragm were increased in male rats exposed to 2 mg/m^3^ MWNT-7. In female rats exposed to 2 mg/m^3^ MWNT-7, there was a non-significant increase in the incidences of simple mesothelial hyperplasia and focal fibrosis of the parietal pleura. The incidence of focal fibrosis of the ventral pleura was elevated in male and female rats exposed to 2 mg/m^3^ MWNT-7. Finally, the incidence of inflammation of the mediastinum was elevated in male rats exposed to 2 mg/m^3^ MWNT-7.

Goblet cell hyperplasia was also observed in the frontal respiratory epithelium of the nasal cavity and nasopharynx of male and female rats exposed to 0.2 and 2 mg/m^3^ MWNT-7 and in the respiratory epithelium of the nasopharynx of female rats exposed to 0.02 mg/m^3^ MWNT-7. Incidences of eosinophilic globules, which appeared in the cytoplasm of the respiratory and olfactory epithelia in the nasal cavity, were increased in male and female rats exposed to 0.2 and 2 mg/m^3^ MWNT-7. Eosinophilic globules could be the normal reaction to mild irritation [24]. No remarkable non-neoplastic changes were found in the other organs.

Microscopically, single or aggregated MWNT-7 s were found in the nasal cavity, larynx, trachea, lungs, lymph nodes, spleen, liver, kidneys, olfactory bulb, and brain of the exposed rats of both sexes. MWNT-7 fibers in the kidney, olfactory bulb, and brain were single and not aggregated. The degree of MWNT-7 deposition in the lungs and mediastinal lymph nodes was concentration-dependent. Little MWNT-7 was observed by light microscopy outside of the lungs and mediastinal lymph nodes.

### Cytological and biochemical analyses of bronchoalveolar lavage fluid (BALF)

**Fig. 4:**
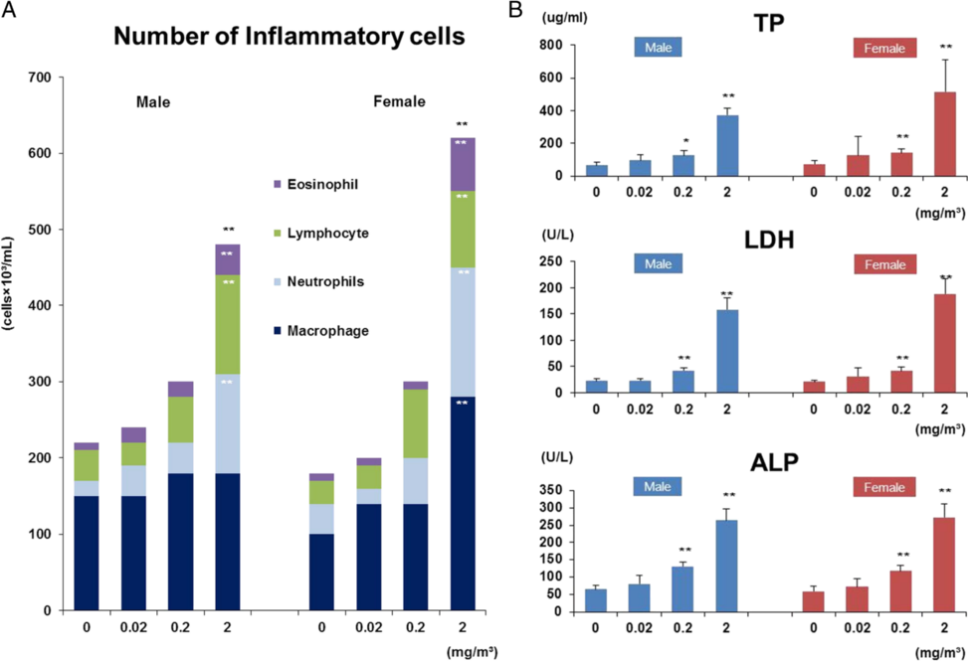
Cytological and biochemical analyses of bronchoalveolar lavage fluid (BALF). **a** Cytological analyses. Total cells per 1 ml in the BALF is shown. There is a concentration-dependent increase in the numbers of inflammatory cells, and the numbers of eosinophils, lymphocytes, neutrophils, and macrophages in the males and females exposed to 2 mg/m^3^ MWNT-7 are significantly increased. **: *p* < 0.01 by Dunnett’s multiple comparison test. **b** Biochemical analyses: Total protein (Tp), Lactate dehydrogenase (LDH), and alkaline phosphatase (ALP) levels in the BALF were determined for male and female rats. Error bars indicate the SD for 5 rats. Tp, LDH, and ALP are significantly increased in a concentration-dependent manner in males and females. *: *p* < 0.05 and **: *p* < 0.01 by Dunnett’s multiple comparison test

The numbers of neutrophils, eosinophils, lymphocytes, and macrophages were increased in the BALF of males and females concentration-dependently and neutrophils, eosinophils, and lymphocytes were significantly elevated in 2 mg/m^3^ MWNT-7 exposed males and females, and macrophages were also significantly higher in 2 mg/m^3^ MWNT-7 exposed females (Fig. 4a). ALP, LDH, and total protein levels were also elevated in the BALF of males and females concentration-dependently and were significantly elevated in males and females exposed to 0.2 and 2 mg/m^3^ MWNT-7 (Fig. 4b).

### MWNT-7 lung burden

**Fig. 5:**
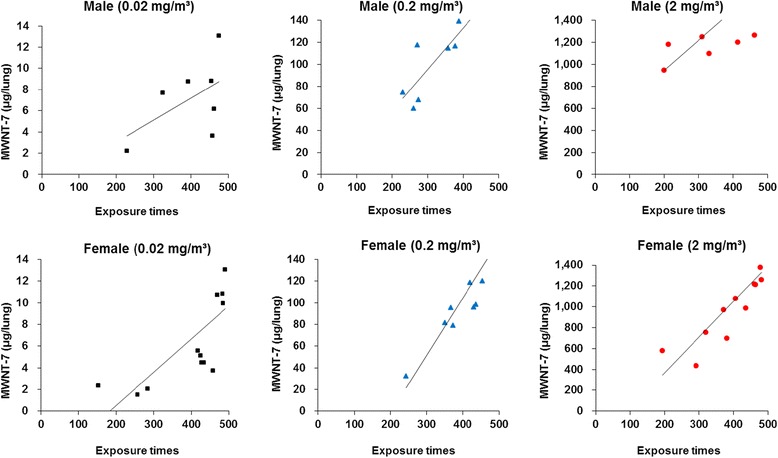
Results of lung burden analysis. MWNT-7 fibers in the lung were measured for rats that died or were sacrificed in a moribund condition before the end of the experiment. The lung burden increased with concentration and time of exposure

**Fig. 6:**
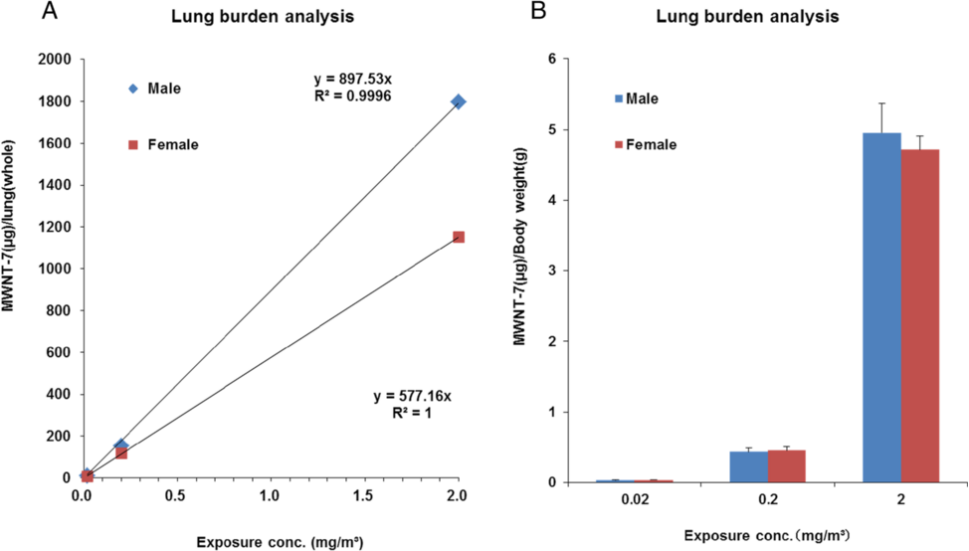
Results of lung burden analysis in rats at 104 weeks. The lung burdens of 10 rats from each exposure group were measured. **a** Total amounts of MWNT-7 (μg) in whole lungs in male and female rats is plotted against the exposure concentration. The amount of MWNT-7 in the lungs increased linearly in a concentration-dependent manner. **b** The relative amount of MWNT-7 (μg/body weight) in the lungs of male and female rats. The relative amounts of MWNT-7 found in the lungs are the same in males and females

Figure 5 shows the MWNT-7 lung burden of the rats that were euthanized or died before the end of the 104-week experimental period. The amount of retained MWNT-7 was related to the MWNT-7 concentration and overall exposure time. The results of the lung burden analysis of MWNT-7 at the end of exposure period are presented in Fig. 6. Amounts of MWNT-7 in the lungs increased linearly with concentration in both males and females (Fig. 6a). The relative amounts of MWNT-7 per body weight were similar between the males and females in the different exposure groups (Fig. 6b).

A summary of the incidence of lung carcinoma and the lung burden of MWNT-7 in the exposed groups at the end of the 104-week experimental period is given in Table 4. One μg of MWNT-7 was determined to equal 9.03 × 10^6^ MWNT-7 fibers by SEM examination. The numbers of MWNT-7 fibers in the lungs of each exposure group was calculated based on this number of MWNT-7 fibers per μg and the result of the lung burden analysis. The number of MWNT-7 fibers in the lung increased in a concentration dependent manner (Table 4). The number of fibers per body weight were similar between males and females in the different concentration groups. The number of fibers that induced lung carcinoma was calculated to be 1.38 × 10^9^ MWNT-7 fibers in 0.2 mg/m^3^ MWNT-7 exposed males and 10.4 × 10^9^ MWNT-7 fibers in 2 mg/m^3^ MWNT-7 exposed females. The number of fibers per gram body weight that induced lung carcinoma was calculated to be 3.92 × 10^6^ MWNT-7 fibers/gram body weight in males and 42.5 × 10^6^ MWNT-7 fibers/gram body weight in females.

**Table 4:** Incidence of lung carcinoma and lung burden in rats exposed to MWNT-7 for 104 weeks

Concentration (mg/m^3^)	Lung carcinoma incidence (%)		Lung burden		
		Amount of MWNT-7 (ug/lung)	Number of MWNT-7 (fibers/lung)	Amount of MWNT-7 (ug/body weight:g)	Number of MWNT-7 (fibers/body weight:g)
Male					
0	2	-	-	-	-
0.02	2	10.0	0.09 × 10^9^	0.029	0.26 × 10^6^
0.2	16 *	152.4	1.38 × 10^9^	0.434	3.92 × 10^6^
2	22 **	1797.8	16.2 × 10^9^	4.954	44.7 × 10^6^
Female					
0	0	-	-	-	-
0.02	2	8.1	0.07 × 10^9^	0.034	0.31 × 10^6^
0.2	0	118.4	1.07 × 10^9^	0.453	4.09 × 10^6^
2	16 **	1154.1	10.4 × 10^9^	4.712	42.5 × 10^6^

Number of MWNT-7: 1ug = 9.03 × 10^6^ (calculated by SEM)

Significant difference; *: *p*≦0.05 **: *p*≦0.01 by Fisher Exact test

### Observation of MWNT-7 in the lung

**Fig. 7:**
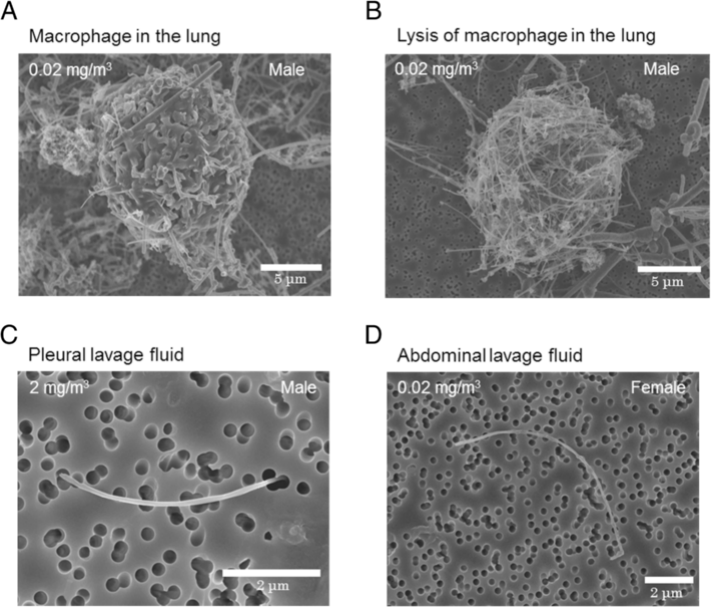
SEM image of alveolar macrophages and MWNT-7 in the lung, and MWNT-7 in the pleural or abdominal fluid. **a** An incompletely digestion alveolar macrophage from the lung of a male rat exposed to 0.02 mg/m^3^ MWNT-7. **b** After complete digestion of macrophages, large cocoon-like masses of fibers were observed. The spacemen was collected from the lung of a male rat exposed to 0.02 mg/m^3^ MWNT-7. **c** MWNT-7 from the pleural fluid. The morphology of the MWNT-7 fiber is single and straight. **d** MWNT-7 from the abdominal fluid. The morphology of the MWNT-7 fiber is single and straight

To observe MWNT-7 in the lung, lung tissues were digested, and specimens were observed by SEM. Alveolar macrophages which were digested completely or incompletely were obtained from lung tissues. Figure 7a is an incompletely digested macrophage phagocytosing MWNT-7, while Fig. 7b shows a completely digested macrophage. Figure 7b shows a cocoon-like mass approximately 12 μm in diameter formed by MWNT-7 fibers. The fibers in these masses wound around each other to form a dense aggregate. The size of the MWNT-7 masses was approximately 10–20 μm in diameter, and the number of the masses present in the lungs increased concentration-dependently (data not shown). These large aggregates of MWNT-7 fibers came from alveolar macrophages, because MWNT-7 aggregates were also observed in incompletely digested alveolar macrophages. Importantly, these aggregates of MWNT-7 fibers were not observed in the MWNT-7 aerosols in any of the inhalation chambers.

### Measurement of the number MWNT-7 in pleural and abdominal lavage fluid

MWNT-7 fibers were present in pleural and abdominal lavage fluid. The number of MWNT-7 fibers in the pleural area were 38, 134, and 1468 fibers in the 0.02, 0.2, and 2 mg/m^3^ exposed males and 23, 240, and 847 fibers in the 0.02, 0.2, and 2 mg/m^3^ exposed females. The number of MWNT-7 fibers in the abdominal area were 16, 161, and 2429 fibers in the 0.02, 0.2, and 2 mg/m^3^ exposed males and 34, 294, and 3329 fibers in the 0.02, 0.2, and 2 mg/m^3^ exposed females. SEM examination revealed that in the pleural and the abdominal lavage fluid the MWNT-7 were individual, long, straight fibers and not aggregated (Fig. 7c and d).

## Discussion

To test for potential hazard to humans of airborne straight, fibrous MWCNT, rats were exposed to MWNT-7 aerosol for 6 h/day, 5 days/week for 104 weeks. Airborne MWNT-7 showed clear lung carcinogenicity.

Exposure to MWNT-7 aerosol for 104 weeks was found to cause statistically significant increases in the incidences of lung carcinomas and combined adenomas and carcinomas in males exposed to 0.2 and 2 mg/m^3^ MWNT-7 and in females exposed to 2 mg/m^3^ MWNT-7. The increased incidence of lung carcinomas and combined adenomas and carcinomas in males was clearly does-dependent. In both males and females, the lung carcinomas were mainly bronchiolo-alveolar carcinomas. In addition to bronchiolo-alveolar carcinomas, adenosquamous carcinomas, a squamous cell carcinoma, and a poorly differentiated adenocarcinoma were found in 1 male and 3 female rats exposed to 2 mg/m^3^ MWNT-7; these carcinomas very seldom develop spontaneously and are thus likely to be linked to MWNT-7 exposure. In addition, pre-neoplastic lesions (bronchiolo-alveolar hyperplasia and atypical hyperplasia [25, 26]) and reactive hyperplasia (alveolar hyperplasia and bronchial hyperplasia [25, 27]) in the lungs of male and female rats were significantly increased in a concentration-dependent manner. These results are consistent with the development of lung carcinoma.

In the present study, there were no abnormal clinical signs, suppression of body weight gain or delay of growth rate, or decreased survival rate in the exposed groups. Therefore, the highest exposure concentration, 2 mg/m^3^ MWNT-7, fulfills the maximum tolerated dose (MTD) criteria [28, 29] for 104-week bioassay studies of rodent carcinogenicity: the highest dose of the carcinogenicity study should not alter the animals’ normal longevity from toxic effects other than carcinogenicity, and there should be no more than a 10 % weight decrement compared to the concurrent controls.

The fiber paradigm theorizes that longer fibers exert stronger toxic and carcinogenic effects than shorter ones because alveolar macrophages are unable to completely take up longer fibers [30]. In support of this theory, Muller et al. [31] reported that no mesotheliomas were observed in Wistar rats after intraperitoneal injection of MWCNT of average length less than 1 μm. Our results support this length hypothesis: the mean length of MWNT-7 collected from the lungs of exposed rats in the present study was 5.8–5.9 μm.

In the present inhalation study, the number of MWNT-7 fibers in the lung needed for inducing lung carcinoma was over 1 × 10^9^ fibers. Rittinghausen et al. [2] reported intraperitoneal injection of 1 × 10^9^ or 5 × 10^9^ MWCNT fibers resulted in tumor incidences of 40 and 100 %, respectively. These two results suggest that large amounts of MWCNT are needed to induce the development of malignant tumors.

Total lung burden (Deposited lung dose) is calculated as Exposure concentration × Minute ventilation × Exposure duration × Alveolar deposition fraction. The MWNT-7 deposited in the lungs of male rats exposed to 0.02, 0.2, and 2 mg/m^3^ MWNT-7 resulted in total lung burdens of approximately 0.01, 0.15, and 1.8 mg (Table 4), respectively. Using an average minute ventilation for male rats of 186 ml/min [32] and an exposure duration of 6 h/day × 492 days, the deposition fraction was calculated as roughly 1.5–2.7 %.

To begin to assess possible effects of human exposure to MWNT-7 in the workplace, we calculated the correlation of rodent to human exposure in accordance with the report of Kuempel and Erdely [33, 34]: Human lung burden equals rat lung burden (0.01, 0.15, and 1.8 mg) × Human alveolar surface area (102 m^2^) / rat alveolar surface area (0.4 m^2^), resulting in approximate equivalent whole lung burdens of 2.6, 38.3, and 459 mg in humans. Assuming (1) human ventilation is 20 L/min during light work, (2) exposure for 8 h per day, 5 days per week, 52 weeks per year, over a working lifetime of 45 years, and (3) a deposition efficiency of 4 %, then lung burdens of 2.6, 38.3, and 459 mg in humans would require that workers are exposed to concentrations of 0.6, 8.5, and 102.2 μg/m^3^ MWNT-7: human lung burden equals (airborne MWNT-7 concentration) × (20 L/min × 10^-3^ m^3^/L × 60 min /h × 8 h/d × 5 d/week × 52 weeks/year × 45 years) × 4 %. Of note, a worker exposed to a concentration of 8.5 μg/m^3^ can expect an approximate lung deposition of 38.3 mg, which is equivalent to a lung burden of 150 μg in rats, a lung burden that resulted in development of lung carcinoma in male rats. Values of 0.6, 8.5, and 102.2 μg/m^3^ are within the range of reported workplace exposures with measured total gravimetric mass concentrations of MWCNT ranging from 0.7 to 332 μg/m^3^ in personal breathing zones [35–37].

The amount of MWNT-7 retained in the lungs increased with concentration, and the development of pre-neoplastic and neoplastic lesions in the lungs also increased with concentration. This is consistent with the premise that biopersistence is an important factor in fiber-dependent generation of neoplasias [30]. This may also explain the requirement for the large amounts of MWCNT that are needed to induce the development of malignant tumors.

In our previous study, we observed large aggregates of MWNT-7 in alveolar macrophages [21, 22], and in the present study large aggregates of MWNT-7 were also observed in digested alveolar macrophages. These aggregates were composed of MWNT-7 fibers that wound around each other forming large dense cocoon-like shaped masses approximately 10–20 μm in diameter (Fig. 7b). The formation of these masses is most likely due to the structural flexibility of MWNT-7. There was a concentration-dependent increase in the number of these masses in the lung tissue. The increase of undigested MWNT-7 s with increased exposure time demonstrates the possibility of a continuous, extended reaction against these fibers: the persistence of phagocytosed indigestable materials have been reported to be associated with production of high levels of ROS and inflammatory cytokines [38–41]. In addition, Donaldson et al. [30] reported that frustrated phagocytosis by macrophages attempting to enclose long fibers leads to prolonged release of cytokines and oxidants.

The presence of large aggregates of MWNT-7 fibers in alveolar macrophages is likely to be a source of intracellular stress, even if they do not explicitly demonstrate incomplete phagocytosis (Fig. 7b). Fractured alveolar macrophages in the proximal alveolar space were also found in the present study. Our results suggest that not only the length but also the quantity of MWNT-7 fibers is important in the pulmonary toxicity of MWNT-7. Overall, our results support the paradigm that biopersistence of fibers such as MWNT-7 leads to persistent generation of oxidants and cytokines, which in turn leads to continuous inflammation, and that inflammation can lead to the development of hyperplastic lesions (Table 3) that have the potential to develop into malignant tumors (Table 3).

There was a gender difference in the development of pre-neoplastic and neoplastic lesions in MWNT-7-exposed rats: exposure to 0.2 mg/m^3^ MWNT-7 resulted in development of lung tumors in males but not females. The lung weights of the males and females were 1.51 and 1.14 g, respectively (Table 2), and the number of MWNT-7 fibers in the whole lung of males and females exposed to 0.2 mg/m^3^ MWNT-7 was 1.38 × 10^9^ and 1.07 × 10^9^ fibers, respectively (Table 4). Consequently, the number of MWNT-7 fibers per lung weight (g) in males and females was 0.91 × 10^9^ and 0.94 × 10^9^, respectively. These results indicate that there was not a relationship between the number of fibers per gram of lung and the gender difference in tumor development in the present study. However, the historical control data our institute has complied from the control groups of inhalation carcinogenicity studies conducted over the past 10 years, shows that the incidence of spontaneous bronchiolo-alveolar tumors in F344 rats is higher in males than in females (bronchiolo-alveolar adenoma: 35/599 males and 14/600 females, bronchiolo-alveolar carcinoma: 6/599 males and 0/550 females). Therefore, the difference in the sensitivity between males and females exposed to MWNT-7 is likely to be due to gender differences in their basic susceptibility to the development of lung tumors.

Sargent et al. [19] reported that MWNT-7 inhalation exposure at 5 mg/m^3^ for 15 days using mice pretreated with methylcholanthrene (a tumor initiator) increased incidences of bronchiolo-alveolar tumors in the lungs at 17 months post-exposure. However, exposure of MWNT-7 without an initiator did not increase the incidences of lung tumors in their study. Consequently, they concluded that MWNT-7 s have a promoter effect in mouse lung carcinogenesis. In contrast, in our study, exposure of rats to MWNT-7 for 104 weeks without a tumor initiator induced development of lung carcinomas. These two results suggest that not only amounts of MWNT-7 but also exposure duration have important roles in lung carcinogenesis mediated by MWNT-7. Additionally, it is likely that the mouse is less susceptible to particle induced carcinogenesis than the rat, as previous investigations of rodent exposure have shown that exposure to asbestos by inhalation or intratracheal instillation is strongly carcinogenic in the rat lung but only weakly carcinogenic in the mouse [42–44].

There was no induction of pleural mesothelioma, however, there was a concentration-dependent increase of proliferative lesions, such as mesothelial hyperplasia in the parietal pleura, in both males and females. Notably, the number of MWNT-7 fibers in the pleura of the 2 mg/m^3^-MWNT-7 exposed groups was 1 × 10^3^ fibers, 10^6^ fold lower than the number of fibers needed to result in tumor formation in the lung. The considerable difference in fiber number between the lung and the pleura likely accounts for the difference in tumor induction in these organs. Recently it was reported that pleural mesotheliomas were induced in rats administered MWCNT (NIKKISO, Tokyo, Japan) at a dose of 1 mg over a 2-week period by intratracheal instillation and then followed for 2 years without any treatment [45]. Further studies are required to determine the reason for the different results obtained from administration using inhalation and intratracheal instillation.

Straight MWCNT fibers and asbestos are thought to have similar physical characteristics, such as a long and thin form (i.e., high aspect ratio) and high mechanical strength, although the chemical surface properties are different. Male rats exposed to asbestos by inhalation developed lung tumors and mesothelioma [46, 47]. Histopathological classification of the lung tumors were adenoma, adenocarcinoma, squamous cell carcinoma, and unclassified lung carcinoma. Biopersistence of asbestos fibers is a primary factor in the toxic effects exerted by asbestos [30, 48]. The results of asbestos studies and our study point to a similarity between MWNT-7 and asbestos hazard, supporting previous predictions of the risk of MWCNT fibers to exposed humans [30].

The determination of whether a genotoxic or non-genotoxic mechanism operates in MWNT-7 induced carcinogenicity is important for carcinogen risk assessment. In an in vitro study, Ames tests of MWNT-7 were negative, indicating that MWNT-7 is not a direct DNA mutagen [49]. In contrast, using eukaryotic cells positive results for MWNT-7 genotoxicity were obtained: Ema et al. [49] and Asakura et al. [50] reported that MWNT-7 genotoxicity was characterized by formation of polyploidy without structural chromosomal aberration; Asakura et al. [50] reported increased numbers of bi- and multi-nucleated cells without micronucleus induction; Yasui et al. [51] observed that comparatively long MWNT-7 (approximately ≥ 20 μm) inhibited cytokinesis during cell division and induced the formation of binucleated cells, whereas short MWNT-7 did not. Interestingly, Kato et al. [52] reported that MWNT-7 induced micronucleus formation and sister chromatid exchange. Taken together, these results indicate that MWNT-7 does not induce mutations through direct interaction with the DNA (negative results in Ames tests) but rather through direct interference with biological processes during cytokinesis. In addition, Kato et al. [52] report that intratracheal instillation of MWNT-7 in mice increased gpt mutation frequencies, DNA damage by comet assay, and DNA oxidative damage. This type of damage is consistent with damage due to oxidative stress and inflammatory responses, but gpt mutations and oxidative damage are not consistent with aberrant cytokinesis. Therefore, these results suggest that the genotoxicity of MWNT-7 in vivo is due to secondary mechanisms such as oxidative stress and inflammatory responses.

Because of the differences in fiber length, shape, and rigidity of different MWCNTs, factors that are likely to be crucial in the development of tumors, the present study does not apply to all MWCNTs. However, based on MWNT-7, a fibrous MWCNT, having a carcinogenic potential, it is important to consider the risk to humans of exposure to rigid MWCNTs with a similar length and width.

Based on animal experimental studies, IARC [53] evaluated MWNT-7 as being a possible carcinogen to humans (Group 2B). NIOSH (2013) [54] proposed an 8-h time-weighted average concentration of 1 μg/m^3^ as the Recommended Exposure Limit (REL) for the respirable mass fraction of elemental carbon, single or multi-walled carbon nanotubes, and carbon nanofibers.

For risk analysis, a point of departure (PoD) from the observed carcinogenesis data should be estimated to mark the beginning of extrapolation to lower doses. The lower confidence limits of the benchmark dose yielding a response with a 10 % extra risk (BMCL_10_) were calculated with US.EPA’s benchmark dose software Version 2.6 (US.EPA, 2015) [55]. In the present study, induction of lung carcinoma in male rats was observed at 0.2 mg/m^3^ MWNT-7 and the BMCL_10_ of adverse effect in exposed male rats was calculated to be 0.01 mg/m^3^.

MWNT-7 is classified as a genotoxicic substance but not directively DNA-reactive [49–52], and it has been accepted that there are thresholds for carcinogens that induce mutations via secondary genotoxic mechanisms [56, 57]. Consequently, MWNT-7 may be considered to have a threshold of carcinogenicity.

The no observed adverse effect level (NOAEL) for carcinogenicity of MWNT-7 in this study was 0.02 mg/m^3^ since exposure to this level of MWNT-7 did not result in the development of lung carcinomas in male rats. The chronic toxicity of MWNT-7, the low-observed-adverse-effect level (LOAEL), was 0.02 mg/m^3^ since absolute and relative lung weights were significantly elevated in female rats exposed to 0.02 mg/m^3^ MWNT-7.

## Conclusions

There is clear evidence of MWNT-7 carcinogenicity in male and female F344 rats exposed to MWNT-7 aerosol by inhalation for 104 weeks. Lung carcinomas were significantly increased in male rats by exposure to 0.2  and 2 mg/m^3^ MWNT-7 and in female rats by exposure to 2 mg/m^3^ MWNT-7. Combined adenoma and carcinoma in the lung were also significantly increased by exposure of male rats to 0.2 and 2 mg/m^3^ MWNT-7 and by exposure of female rats to 2 mg/m^3^ MWNT-7. Finally, pre-neoplastic epithelial lesions were also significantly increased by exposure of male rats to 0.2 and 2 mg/m^3^ MWNT-7 and by exposure of female rats to 2 mg/m^3^ MWNT-7. The induction of carcinomas and combined carcinomas and adenomas was dose-dependent in male rats, and the induction of pre-neoplastic epithelial lesions was dose-dependent in both males and females.

Induction of plural mesothelioma by exposure to MWNT-7 was not observed in this study. However, simple mesothelial hyperplasia and focal fibrosis in the parietal pleura were found in rats exposed to 2 mg/m^3^ MWNT-7.

In the studies reported to date, including this one, three factors stand out as crucial for induction of tumors by exposure to MWNT-7: (1) length, (2) biopersistence, and (3) quantity of fibers in the exposed tissue. One likely factor in the lack of mesothelioma development in the MWNT-7 exposed rats is the low number of MWNT-7 fibers in the pleura: 1.38 × 10^9^ and 10.4 × 10^9^ fibers were found in the lungs of the 0.2 mg/m^3^ MWNT-7 exposed males and the 2 mg/m^3^ MWNT-7 exposed females, respectively, while only on the order of 1 × 10^3^ fibers were found in the pleura of the 2 mg/m^3^ MWNT-7 exposed animals.

## Methods

### Test substance

The MWCNT (MWNT-7; Lot No. 080126 until 88 weeks, Lot No. 071223 from 89 weeks) was purchased from Hodogaya Chemical, Co. Ltd. (Tokyo, Japan). This MWNT-7 was used without further purification or sieving. According to Hodogaya Chemical, Co. Ltd, the MWNT-7 fibers were generated using a floating chemical vapor deposition (CVD) process with a carbon purity of > 99.6 % (No. 071223) and > 99.8 % (No. 080126), a nominal average diameter of 40 to 90 nm, an aspect ratio of greater than 100, and a surface area of 24–28 m^2^/g. We previously reported our analysis of MWNT-7 using LA-ICP-MAS: iron, chromium and nickel in MWNT-7 were 4400, 48, and 17 ppm, respectively [58].

The length and width of bulk MWNT-7, the MWNT-7 in the inhalation chambers, and the MWNT-7 in the lungs was measured using SEM at magnifications of × 5000 and × 10,000 in our institute. The lengths of 1000 and the widths of 500 bulk MWNT-7 fibers were measured using a curvimeter and scale loupe on enlarged SEM-photographs. The lengths and widths of 200 MWNT-7 fibers collected from the inhalation chambers and 200 MWNT-7 fibers collected from the lungs at the end of the experimental period were measured using the SEM’s image analysis software. Bulk MWNT-7, lot No. 071223, fibers had an average width of 83.8 nm and length of 5.2 μm, with 45.1 % of the tubules being longer than 5 μm. Bulk MWNT-7, lot No. 080126, fibers had an average width of 90.7 nm and length of 5.7 μm, with 48.7 % of the tubules being longer than 5 μm. The MWNT-7 fibers collected from the inhalation chamber had an average with of 92.9–98.2 nm and length of 5.4–5.9 μm. The MWNT-7 fibers found in the lung had an average width of 95.5–109.6 nm and length of 5.8–5.9 μm.

### Animals

We used two-hundred F344/DuCrlCrlj rats of each sex purchased from Charles River Japan Inc. (Kanagawa, Japan) at 4 weeks of age. The animals were quarantined and acclimated for 2 weeks before the commencement of the experiment (exposure started at 6 weeks of age). The animals were divided by stratified randomization into 4 body weight-matched groups of 50 rats of each sex for exposure to 0, 0.02, 0.2, or 2 mg/m^3^ MWNT-7. During the acclimation and exposure periods, rats were housed individually in stainless-steel wire hanging cages (150 W × 216 D × 176 H mm) placed in pyramid-shaped stainless steel inhalation exposure chambers (10 m^3^ in volume). Throughout the experimental period including quarantine, acclimation, and exposure, all animals had free access to sterilized water and gamma-irradiation-sterilized commercial pellet diet (CRF-1, Oriental Yeast Co. Ltd., Tokyo, Japan). Fluorescent lighting was controlled automatically to give a 12-h light/dark cycle.

This study was conducted in accordance with the Organisation for Economic Co-operation and Development (OECD) Guideline for Testing of Chemicals 451 “Carcinogenicity Studies” (OECD, 2009) [23] and with “Standards to be Observed by Testing Institutions” Notification No. 76 of the Ministry of Labour, Japan, 1 September 1988 (amendment: Notification No. 13 of the Ministry of Labour, Japan, 29 March 2000) and with the OECD Principles of Good Laboratory Practice (OECD, 1981) [59]. This study was approved by the Animal Experiment Committee of the Japan Bioassay Research Center. Animal care and was conducted in accordance with the Guideline for proper conduct of Animal Experiments (Science Council of Japan, 2006).

### Environment in the inhalation chamber and MWNT-7 exposure design

Throughout the 104-week experimental period, the ventilation flow rate of the inhalation chamber was maintained at 1.67 m^3^/min, corresponding to 10 changes/hour, and the chamber temperature and humidity were set at 22 ± 2 °C and 50 ± 20 %. The inhalation chamber environmental parameters were monitored every minute with sensors for airflow, temperature (Shimaden, Co. Ltd., Tokyo, Japan), and humidity (NGK Spark Plug, Co., Ltd., Nagoya, Japan).

Groups of 50 rats of each sex were exposed to MWNT-7 aerosol for 104 weeks (6 h/day, 5 days/week) at target concentrations of 0, 0.02, 0.2, or 2 mg/m^3^. Our facility is large enough to accommodate the 400 animals used in this study, therefore all of the rats were exposed at the same time. The MWNT-7 concentrations used in this inhalation exposure study were decided on the basis of the results of our previous 13-week inhalation study using MWNT-7 concentrations of 0, 0.2, 1, and 5 mg/m^3^ [22].

### Aerosol generation and inhalation exposure to MWNT-7

**Fig. 8:**
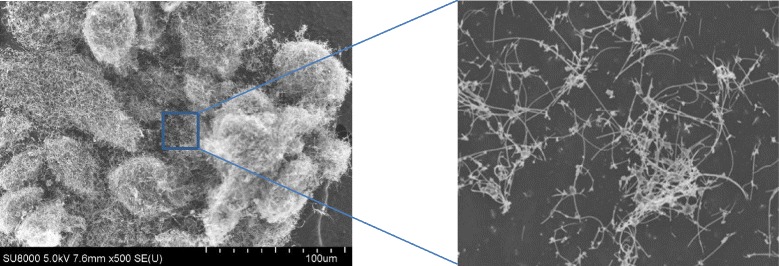
SEM images of bulk MWNT-7. The bulk MWNT-7 is composed primarily of large clumps of MWNT-7. The sizes of the aggregate are around 50 nm in diameter. While less, there are also individual and fibrous MWNT-7 s present in the bulk MWNT-7

The aerosol generation method (cyclone sieve method) of Kasai et al. [20–22] was used to generate MWNT-7 aerosols. We have developed an aerosol generator that dry-aerosolizes MWNT-7 with an upward spiraling airstream, classifies particles by gravitational and centrifugal forces, limits particle size with a sieve, and provides the inhalation chamber with a size-limited MWNT-7 aerosol (Additional file 1: Figure S1). The aerosol generator is composed of an aerosol generation and sieving unit, a concentration control unit, ionizers (Keyence Corporation, Tokyo, Japan), and a touch panel data recording device for data collection. The aerosol generation and sieving unit consists a cylindrical container with a partitioning sieve (Seishin, Co. Ltd., Tokyo, Japan) and a solenoid impactor. The concentration control unit consists of an optical particle controller (OPC, OPC-AP-600, Sibata Scientific Technology, Ltd., Tokyo, Japan) and a dust feeder (Type FO, Funken Powtechs, INC,Tokyo, Japan). The dust feeder introduces bulk-MWNT-7 (Fig. 8) into the mid-portion of the cylindrical container. Clean air is aspirated from nine diagonally-opened slits installed at the bottom of the cylinder by utilizing ejector air with a flow rate of 100 L/minute as the driving force, so that an upward spiraling airstream is continuously generated. Bulk MWNT-7 is dispersed and aerosolized by collision of the MWNT-7 with the high-speed upward spiraling airstream provided from the slits. Shear forces within the cyclone as well as in the ejector also contribute to dispersion. Light MWNT-7 particles are carried to the top of the cylindrical container, where a partitioning sieve is located, riding on the upward spiraling airstream and selectively passing through the pores of the sieve (53 μm). Heavy MWNT-7 particles spiral around under the sieve, agglomerate by the centrifugal force generated by the upward spiraling airstream, and finally fall down into the collection flask. Pulse vibration generated by a solenoid impactor prevents MWNT-7 clogging of the partitioning sieve pores. Ionizers are deployed to prevent adsorption onto walls of the system’s components and to prevent self-assembly of MWNT-7 aerosols.

### Monitoring and regulation of MWNT-7 aerosol in the inhalation chamber

Chamber atmosphere samples were taken from the animals’ breathing zone. Monitoring and regulation of MWNT-7 aerosols in the inhalation exposure chambers is described in detail by Kasai et al. [20–22]. Particle concentrations of MWNT-7 in the chambers were monitored in real-time with an optical particle controller (OPC). The OPC data were collected every 10 s. The mass-concentrations of MWNT-7 were determined gravimetrically by collecting aerosols on Teflon-binder filters for each target exposure concentration after 1, 3, or 5 h of exposure once every 2 weeks throughout the experimental period. The mass per particle (the K value) was calculated based on the particle concentration data (particles/m^3^) collected by the OPC and the gravimetric results (mass/m^3^). Using this K-value, particle concentrations were converted to mass-concentrations every 30 min for each group during the exposure periods. The chamber MWNT-7 concentrations were held constant by controlling the movement of the dust feeder using a feedback loop from the OPC: when the chamber concentration rose above the upper limit of a designated concentration range, the dust feeder stopped supplying MWNT-7 aerosol to the sieving unit and when the chamber concentration fell below the lower limit, the dust feeder resumed supplying MWNT-7 aerosol to the sieving unit. The size distribution and morphology of the MWNT-7 fibers was determined nine times over the course of 13 weeks throughout the experimental period. The size distribution of the fibers was ascertained using a micro-orifice uniform deposit cascade impactor (MOUDI) (Model 125B NanoMoudi-II, MSP, Shoreview, MN, USA), and the morphology of the fibers was examined by SEM (SU8000, Hitachi Ltd., Tokyo, Japan).

### Clinical observations and urinary, hematological, and blood biochemical analyses

The animals were observed daily for clinical signs and mortality. Body weight and food consumption were measured once a week for the first 14 weeks and every 4 weeks thereafter. Urinary parameters were measured in the last week of the 104-week experimental period with Ames Reagent Strips (Multistix, Siemens Healthcare Diagnostics, Tokyo, Japan). At the terminal necropsy, blood was collected for hematology and blood biochemistry from the abdominal aorta under isoflurane anesthesia after overnight fasting. The blood sample was analyzed with an automatic blood cell analyzer (ADVIA120, Siemens Healthcare Diagnostics Inc. Tarrytown, NY, USA) and an automatic analyzer (Hitachi 7080, Hitachi, Ltd., Ibaraki, Japan) for blood biochemistry.

### Organ weights and macroscopic and microscopic examinations

Organs, including the adrenal, testis, ovary, heart, lung (except for lungs collected for BALF analysis), kidney, spleen, liver and brain, were collected at the terminal necropsy and weighed and examined for macroscopic lesions. To avoid contamination of MWNT-7, animals from each administration group were anatomized separately. After macroscopic examination the nasal cavity, nasopharynx, larynx, trachea, lungs, bone marrow, lymph nodes (including lung associated lymph nodes), thymus, spleen, heart, tongue, salivary gland, esophagus, stomach, small intestine, large intestine, liver, pancreas, kidneys, urinary bladder, pituitary gland, thyroid, parathyroid, adrenal glands, testis, epididymis, seminal vesicle, prostate, ovaries, uterus, vagina, mammary gland, brain, spinal cord, peripheral nerve, olfactory bulb, eye, Harderian gland, muscle, bone, and diaphragm (as a part of the parietal pleura) were examined histopathologically. For histopathological examination, the tissues were fixed in 10 % neutral buffered formalin and embedded in paraffin. The right lung was directly fixed by immersion. The left lung including the BALF collected rats was inflated with fixative at a water pressure of 20–25 cm, and then fixed by immersion. The nasal cavity was decalcified in a formic acid-formalin solution and then transversely trimmed at three levels as previously described [21, 60]. Tissue sections of all organs, including the right lung and left lung, of 5 μm in thickness were prepared and stained with hematoxylin and eosin (H & E) (Additional file 2: Table S1). To detect MWNT-7 s deposited in the larynx, trachea, lungs, pleura (diaphragm), lymph nodes, spleen, liver, kidneys, olfactory bulb, and brain, sections were stained with Kernechtrot stain (Merck, Darmstadt, Germany) for 1 min and washed with distilled water for 5 min.

### Cytological and biochemical analyses of the BALF

At the end of the experimental period, five animals from each group were lavaged (Additional file 2: Table S1). The right bronchus was tied with a thread in order to lavage only the left lung. The lung was lavaged 2 times with 5 ml Eagle’s Minimum Essential Medium (MEM, adjusted pH7.2 with 1 N NaOH and 15 mM HEPES without sodium hydrogen carbonate) for males and 4 ml for females at a water pressure of 20–25 cm (the epithelium is peeled by washing with higher pressure or by washing more than twice). The wash-out was collected for cytological and biochemical analysis and the lung tissue was processed for histopathological examination as described above. The cells in the BALF were counted with an automatic cell analyzer (Sysmex fully automated hematology analyzers XT-2000iv). For biochemical analysis, the BALF was centrifuged at 1960 rpm (800 g) at 4 °C for 10 min, and aliquots of the acellular supernatant were examined with an automatic analyzer (Hitachi 7080, Hitachi, Ltd., Ibaraki, Japan). Total protein (TP), lactate dehydrogenase (LDH), and alkaline phosphatase (ALP) levels were measured by conventional biochemical methods. TP was chosen as an indicator of alveolo-capillary permeability while LDH and ALP were used as indicators of general cytotoxicity and type II epithelial cell toxicity, respectively.

### MWNT-7 lung burden

Lung burden analysis was determined for dead and euthanized animals (Additional file 2: Table S1): 7, 7, and 6 males and 12, 8, and 11 females from the 0.02, 0.2, and 2 mg/m^3^ MWNT-7-exposed groups, respectively. At terminal necropsy, lung burden was determined for 10 MWNT-7-exposed animals from each group (Additional file 2: Table S1). Lungs were weighed and then prepared for histopathological examination; remaining right lung tissue (0.04–0.12 g) was used for lung burden analysis. For determination of lung burden, lung tissue was digested according to the method of Kohyama et al. [61], and filtered to obtain dry specimens. MWNT-7 measurement was performed according to the method of Ohnishi et al. [62]. Lung burden was determined as the MWNT-7 value in the analyzed lung tissue multiplied by the ratio of total lung weight/analyzed lung weight. The amounts of MWNT-7 per whole lung and per gram body weight were determined. For determination of the number of MWNT-7 fibers in the lung, one μg bulk MWNT-7 was suspended in 100 ml distilled water containing 0.1 % Tween 80 as a colloidal dispersant and subjected to ultrasonication for 30 min with an ultrasonic homogenizer (VP-30S, 20 kHz, 300 W, TAITEC Co. Ltd, Tokyo, Japan). A polycarbonate membrane filter (Isopore, Millipore, MA, USA) pre-coated with Pt for electron charge avoidance was positioned on a suction filtration apparatus, and 10 μl of the MWNT-7 suspension was placed on the filter and dried by suction. After drying, an area equal to 1 % of whole filter was subjected to field emission SEM. The MWNT-7 fibers on the filter were photographed for counting at a magnification of × 2000. The number of fibers in one-μg MWNT-7 were calculated from the dilution ratio and area ratio of the filter. The total number of MWNT-7 fibers in the lungs was calculated as the number of fibers in one-μg of MWNT-7 multiplied by the μg of MWNT-7 in the lung.

### Observation of MWNT-7 in the lung

To observe MWNT-7 in the lung, lung tissues were digested according to the method of Kohyama et al. [61]. The lung tissues were left for 5 days in the digestion solution. The solution was then filtered and specimens collected on polycarbonate membrane filters (Isopore, Millipore, MA, USA) pre-coated with Pt for electron charge avoidance. The specimens were observed by SEM.

### Measurement of number MWNT-7 pleural and abdominal lavage fluid

At terminal necropsy, the pleural cavity of ten animals was lavaged once with 8 ml MEM, and the abdominal cavity of ten other animals was lavaged once with 40 ml MEM. The washout was collected and centrifuged at 12,000 rpm (3000 g) at 15 °C for 10 min. The supernatant was removed and the pellet was digested according to the method of Kohyama et al. [61]. A polycarbonate membrane filter (Isopore, Millipore, MA, USA) pre-coated with Pt for electron charge avoidance was positioned on a suction filtration apparatus, and MWNT-7 fibers were collected onto the filter. The morphology of the fibers was determined by SEM examination, and the number of MWNT-7 fibers was counted.

### Statistics

Incidences of neoplastic lesions were analyzed for a dose response relationships by Peto’s test [63] and for a significant difference from the clean air-exposed group by Fisher’s exact test. Incidences of non-neoplastic lesions and urinary parameters were analyzed by the Chi-square test. Survival curves were plotted according to the method of Kaplan-Meier [64], and the log-rank test [65] was used to detect statistically significant differences in survival rates between any MWNT-7 exposed groups of either sex and the clean air-exposed group. Body weight, organ weight, food consumption, hematology, blood biochemical parameters, and biochemical and cytological parameters in the BALF were analyzed by Dunnett’s multiple comparison test. Two-tailed tests were used for all statistical analyses except for Peto’s test. *P* values less than 0.05 were considered to be statistically significant.

## Additional files

Additional file 1: Figure S1. Blue arrows indicate the flow of the MWNT-7. Red arrows indicate aerosolization of the MWNT-7 by the upward spiraling airstream. MWNT-7 (Bulk MWNT-7) is first placed into the dust feeder. The dust feeder transports a portion of the MWNT-7 into the sieving chamber (Left panel). In the sieving chamber, clean air is aspirated from 9 diagonally opened slits by using the ejector air as the driving force, so that an upward spiraling airstream is continuously generated in the cylindrical sieving chamber. MWNT-7 is dispersed and aerosolized by the high-speed spiraling air. Light MWNT-7 particles are carried to the top of the sieving chamber, where a partitioning sieve is located. Only the sieved MWNT-7 can be delivered to the inhalation chamber. Ionizers are used to avoid agglomeration. This equipment has a feedback system for keeping the aerosol concentration in the inhalation chamber constant. (PPS 2292 kb)

Additional file 2: Table S1. Number of Rats Examined for Lung Histopathology, BALF and Lung Burden. (XLSX 13 kb)

## Abbreviations

ALP: Alkaline phosphatase
BALF: Bronchoalveolar lavage fluid
BALT: Bronchus-associated lymphoid tissue
CNT: Carbon nanotube
CVD: Chemical vapor deposition
GSD: Geometric standard deviation
H&E: Hematoxylin and eosin
HPLC: High performance liquid chromatography
LDH: Lactate dehydrogenase
MHLW: Ministry of Health, Labour, and Welfare
MMAD: Mass median aerodynamic diameter
MOUDI: Micro-orifice uniform deposit cascade impactor
MTD: Maximum tolerated dose
MWCNT: Multi-walled carbon nanotube
OECD: Organisation for Economic Co-operation and Development
OPC: Optical particle controller
PAH: Polycyclic aromatic hydrocarbon
SEM: Scanning electron microscope
TP: Total protein
