# Supplementary material for: Lung carcinogenicity of inhaled multi-walled carbon nanotube in rats
Source: Part Fibre Toxicol. 2016 Oct 13;13:53. doi: 10.1186/s12989-016-0164-2 (PMC5064785; doi:10.1186/s12989-016-0164-2)
Supplement: Supplementary file 1 — Blue arrows indicate the flow of the MWNT-7. Red arrows indicate aerosolization of the MWNT-7 by the upward spiraling airstream. MWNT-7 (Bulk MWNT-7) is first placed into the dust feeder. The dust feeder transports a portion of the MWNT-7 into the sieving chamber (Left panel). In the sieving chamber, clean air is aspirated from 9 diagonally opened slits by using the ejector air as the driving force, so that an upward spiraling airstream is continuously generated in the cylindrical sieving chamber. MWNT-7 is dispersed and aerosolized by the high-speed spiraling air. Light MWNT-7 particles are carried to the top of the sieving chamber, where a partitioning sieve is located. Only the sieved MWNT-7 can be delivered to the inhalation chamber. Ionizers are used to avoid agglomeration. This equipment has a feedback system for keeping the aerosol concentration in the inhalation chamber constant. (PPS 2292 kb) [file 12989_2016_164_MOESM1_ESM.pps]

## Slide 1
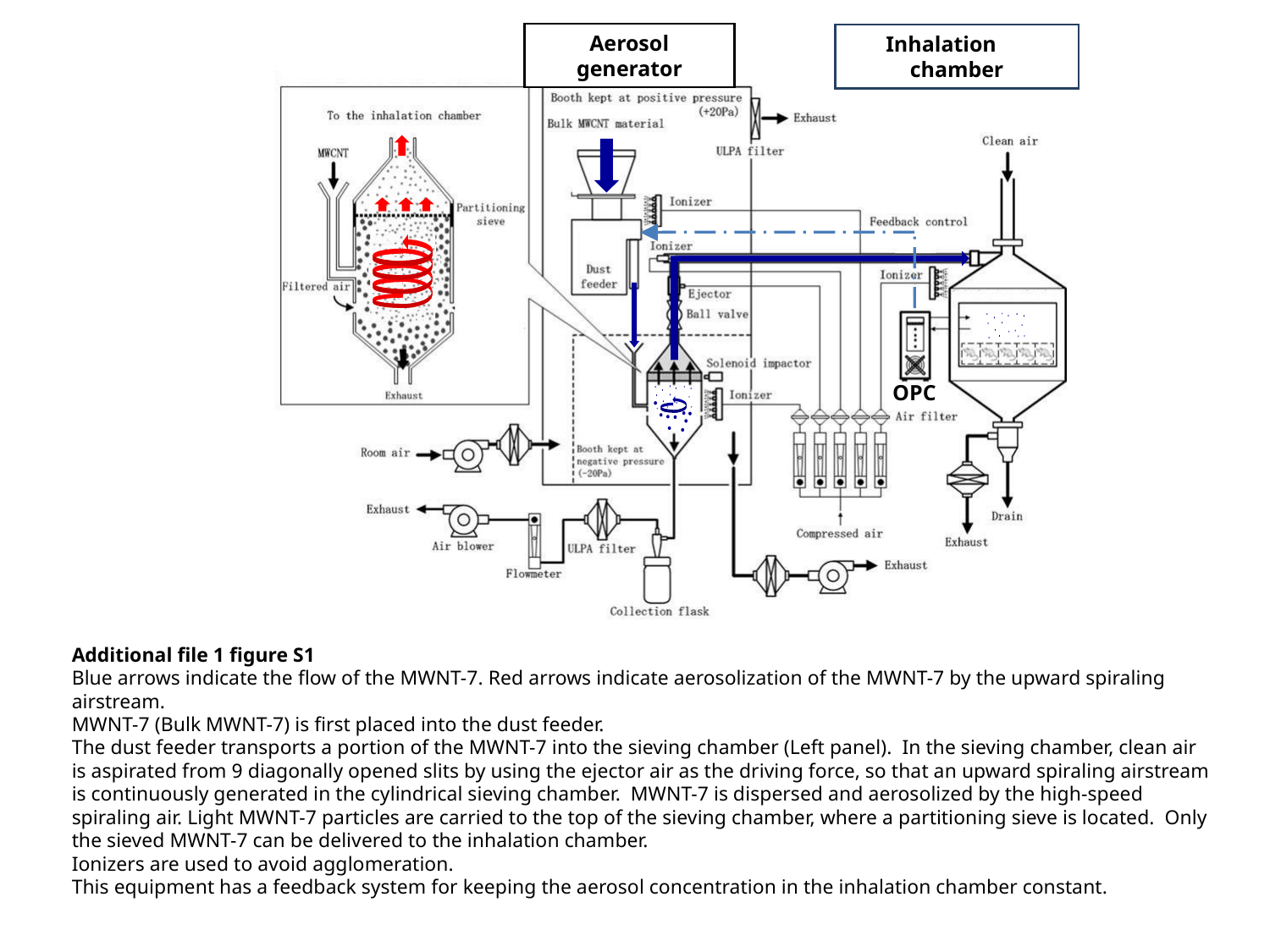

Aerosol generator
Inhalation　chamber
OPC
Additional file 1 figure S1
Blue arrows indicate the flow of the MWNT-7. Red arrows indicate aerosolization of the MWNT-7 by the upward spiraling airstream.
MWNT-7 (Bulk MWNT-7) is first placed into the dust feeder.
The dust feeder transports a portion of the MWNT-7 into the sieving chamber (Left panel). In the sieving chamber, clean air is aspirated from 9 diagonally opened slits by using the ejector air as the driving force, so that an upward spiraling airstream is continuously generated in the cylindrical sieving chamber. MWNT-7 is dispersed and aerosolized by the high-speed spiraling air. Light MWNT-7 particles are carried to the top of the sieving chamber, where a partitioning sieve is located. Only the sieved MWNT-7 can be delivered to the inhalation chamber.
Ionizers are used to avoid agglomeration.
This equipment has a feedback system for keeping the aerosol concentration in the inhalation chamber constant.
